# Towards safer airway management in the critically ill: lessons from National Audit Project 4

**DOI:** 10.1186/cc12093

**Published:** 2013-03-19

**Authors:** N Santana-Vaz, S Tallowin, H Lewis, D Park, R O'Brien, JM Patel

**Affiliations:** 1 Heart of England NHS Foundation Trust, Birmingham, UK

## Introduction

National Audit Project 4 (NAP4) highlighted the need to improve airway management in ICUs and key recommendations were the continuous use of end-tidal carbon dioxide (ETCO_2_) monitoring, pre-intubation checklists and difficult airway trolleys [1]. This complete cycle audit aimed to quantify the current state of airway management on our ICU and the effectiveness of implementing the NAP4 recommendations.

## Methods

Data collection was carried out prospectively for both phases and included documentation of intubation, use of ETCO_2 _and the incidence of serious adverse events (SAEs). The contents of the intubation boxes were compared against the Difficult Airway Society (DAS) guidelines [2]. The re-audit was carried out 6 months following the introduction of a pre-intubation checklist, a documentation sticker, a difficult airway trolley and standardization of the basic bedside airway boxes with a checklist of contents. A training program in airway management for all ICU staff was also introduced.

## Results

The baseline characteristics of both groups were similar. The initial audit included 45 patients and the re-audit 58 patients. In the initial audit 19% of patients had accurate documentation of intubation with the use of ETCO_2_. The continuous use of ETCO_2 _was 80%. Bedside airway boxes did not contain a checklist of standardized equipment. A difficult airway trolley was not located on the ICU. Following introduction of the changes described, a significant improvement in the use of ETCO_2 _to confirm intubation (46% vs. 19%, *P <*0.01) and continuous use of ETCO_2 _monitoring (100% vs. 80% *P *≤0.0004) was observed. The use of the preintubation checklist (7%) and stickers (11%) was poor. All bedside boxes had a checklist and the required equipment. A difficult airway trolley is located on the ICU with an equipment list and DAS algorithm attached. No SAEs were recorded in either phase.

## Conclusion

Serious airway complications are rare. Safer airway management is essential in the ICU to prevent morbidity and mortality. This audit demonstrated that simple measures in conjunction with a structured training program can help to improve the safety of airway management in critically ill patients. Further work is required to improve compliance with the pre-intubation checklist alongside a continued education program.
